# 

**Published:** 1887-12

**Authors:** 


					﻿Vol. VII.
DECEMBER, 1887.
Na 12.
THE
HOMEOPATHIC pHY^lClAfl,
A MONTHLY JOURNAL OF MEDICAL SCIENCE.
“ He is a freeman whom truth makes free, and all are slaves beside.”
“Seek the Truth: come whence it may, cost what it will.”
EDITED BY
Edmund j.	m. d.,
▲ND
WALTER M. JAMES, M. D.
PHILADELPHIA :
No. 1183 8PRITOK STREET.
WEW YOBK
A. L. CHATTERTON & COMPANY, Publisher’s Agents.
T.O3STDO35T»
ALFRED HEATH & CO., Sole Agents fob Great Britain,
114 Ebury Street, Eaton Square.
Yearly Subscription, $2.50, invariably in advance. Single numbers, 95 cts.
Entered at the Philadelphia Port-Office aa Seoond-Claae Mail Matter.
				

